# Supplementary material for: Effectiveness of Mix-and-Match Vaccination in Preventing SARS-CoV-2 Omicron Variant Infection in Taiwan: A Test-Negative Control Study
Source: Vaccines (Basel). 2023 Aug 31;11(9):1441. doi: 10.3390/vaccines11091441 (PMC10535833; doi:10.3390/vaccines11091441)
Supplement: Supplementary file 1 [file vaccines-11-01441-s001.zip › vaccines-2535738-supplementary.pdf]

Supplementary Materials

Supplementary Figures

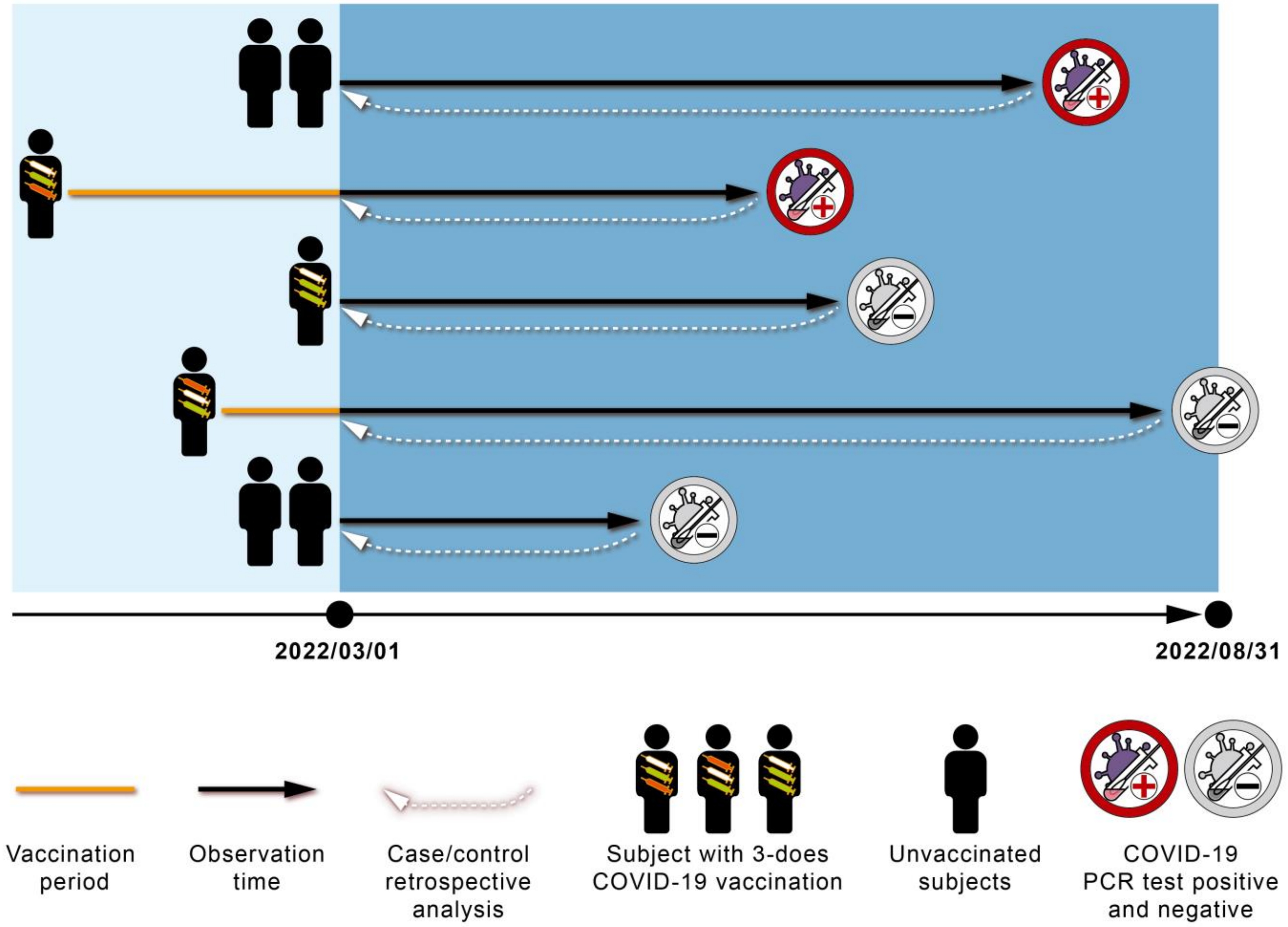

Supplementary Figure S1. Conceptual schematic of study design.

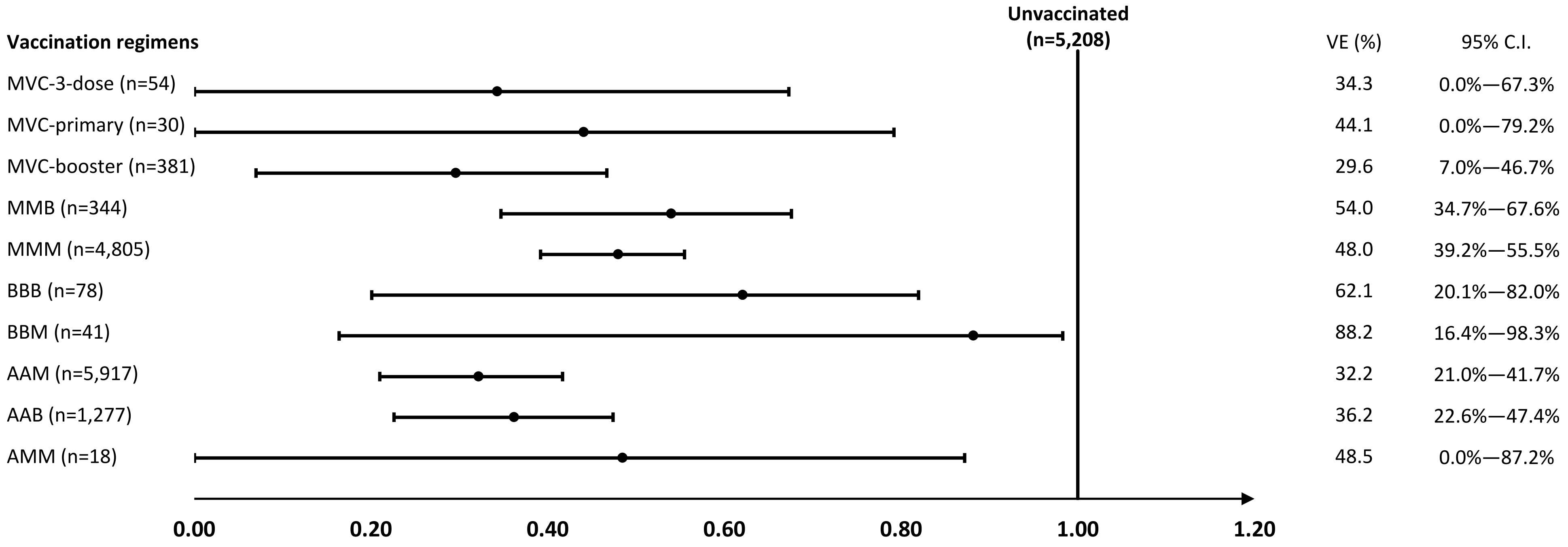

Supplementary Figure S2A. Forest plot of vaccine effectiveness against SARS-CoV-2 infection in persons > 75 years old.

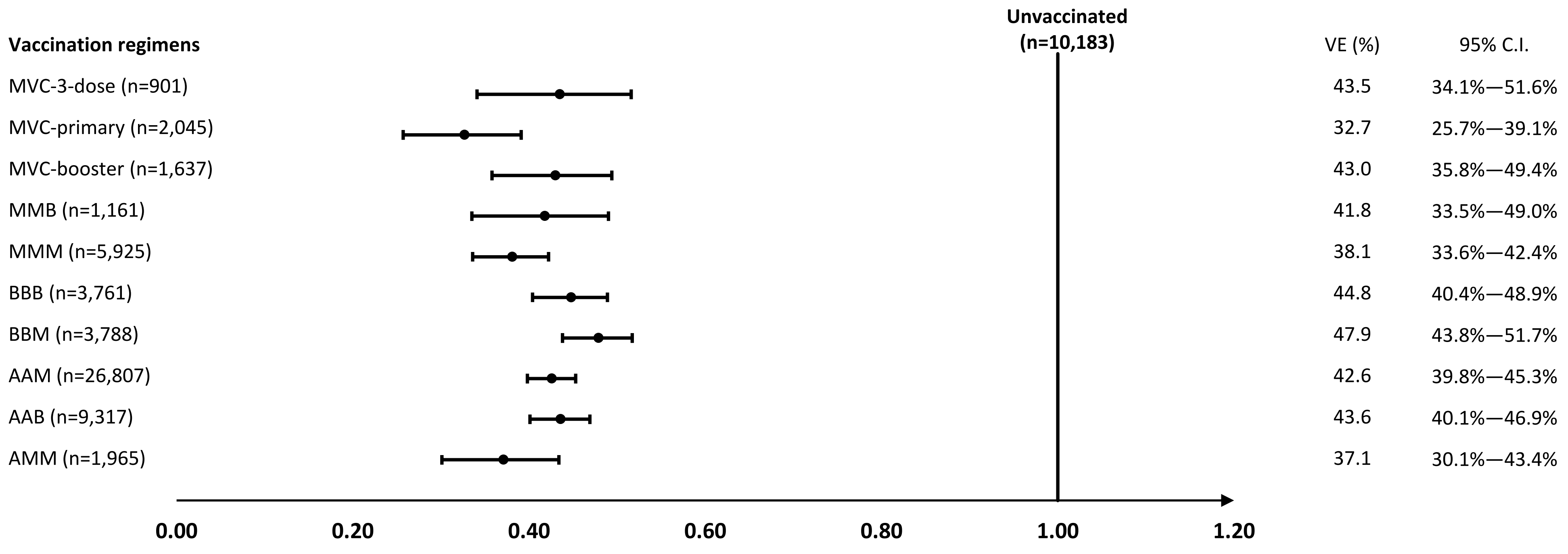

Supplementary Figure S2B. Forest plot of vaccine effectiveness against SARS-CoV-2 infection in persons 20 to 49 years old.

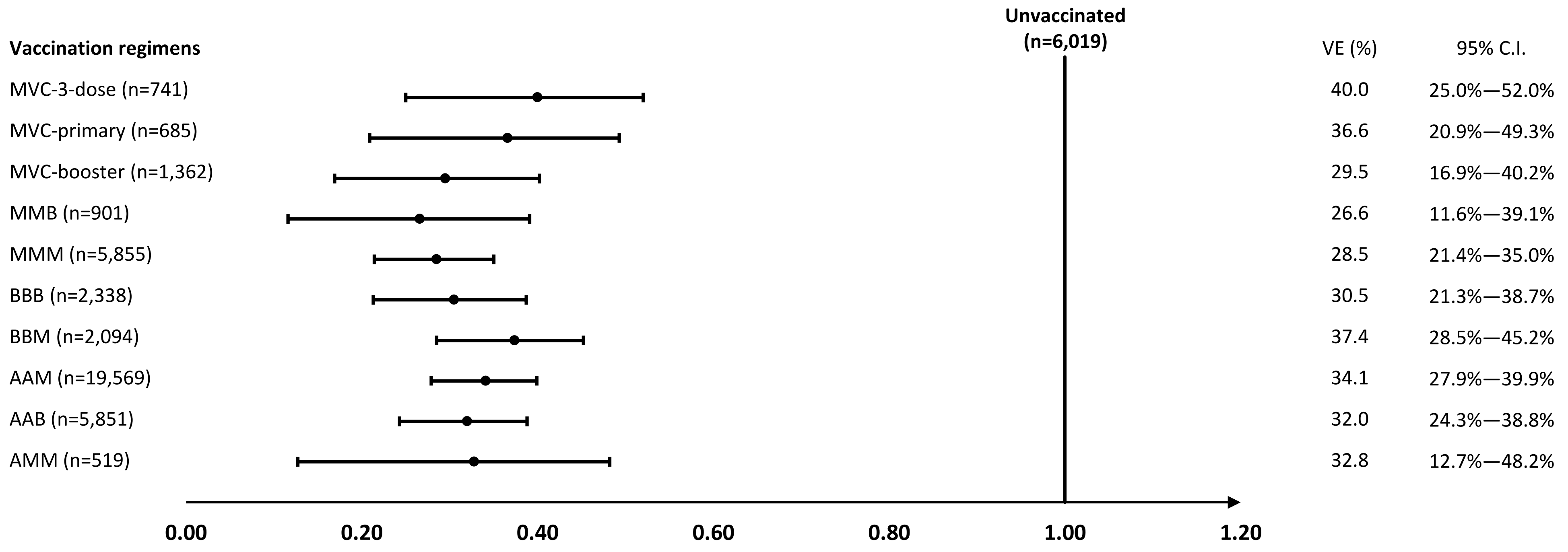

Supplementary Figure S2C. Forest plot of vaccine effectiveness against SARS-CoV-2 infection in persons 50 to 64 years old.

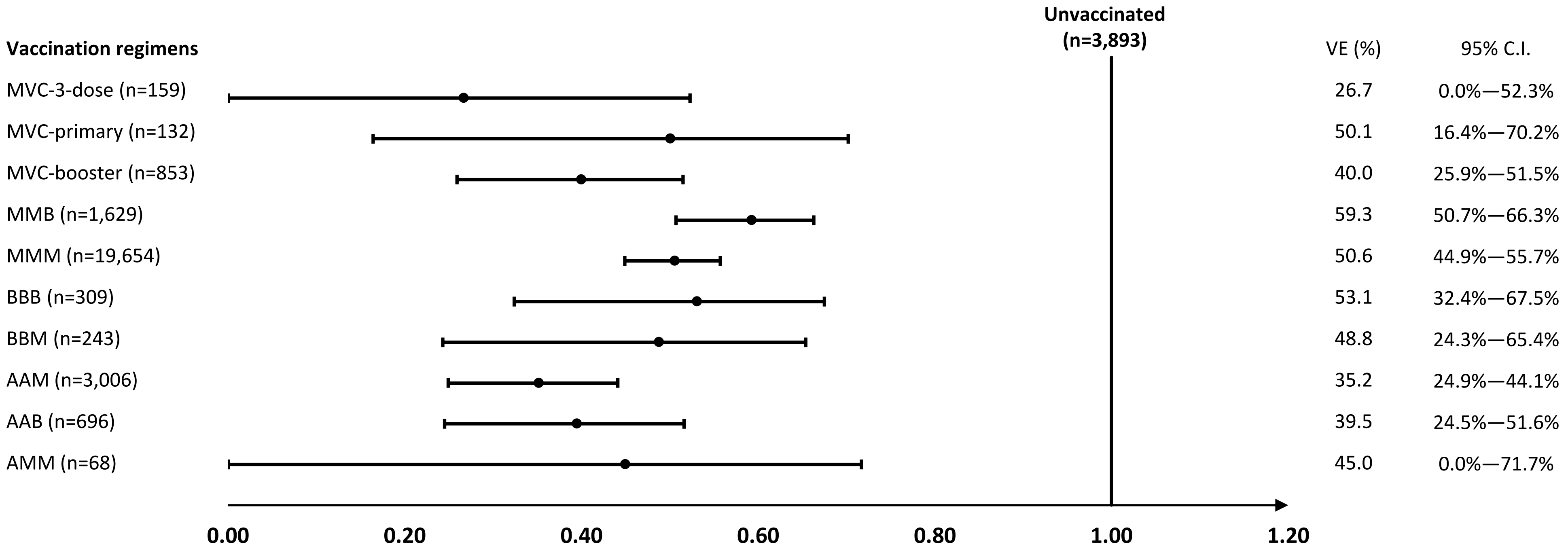

Supplementary Figure S2D. Forest plot of vaccine effectiveness against SARS-CoV-2 infection in persons 65 to 74 years old.

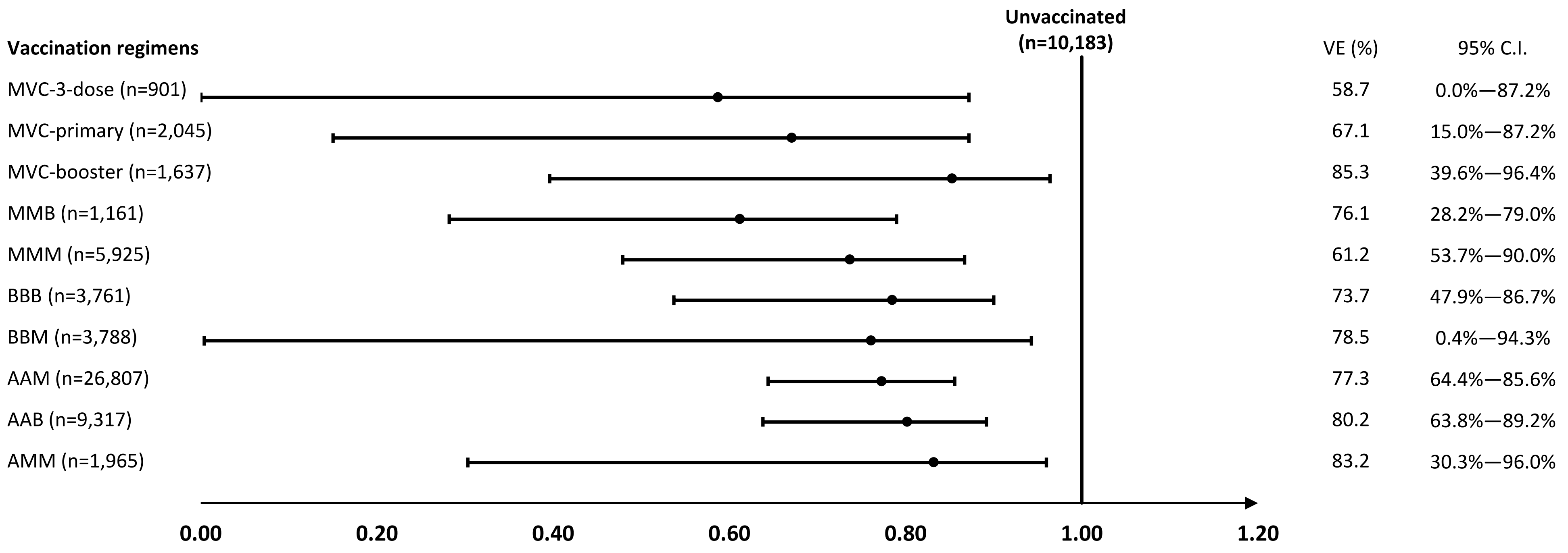

Supplementary Figure S3A. Forest plot of vaccine effectiveness against COVID-19 associated moderate to severe disease in persons 20 to 49 years old.

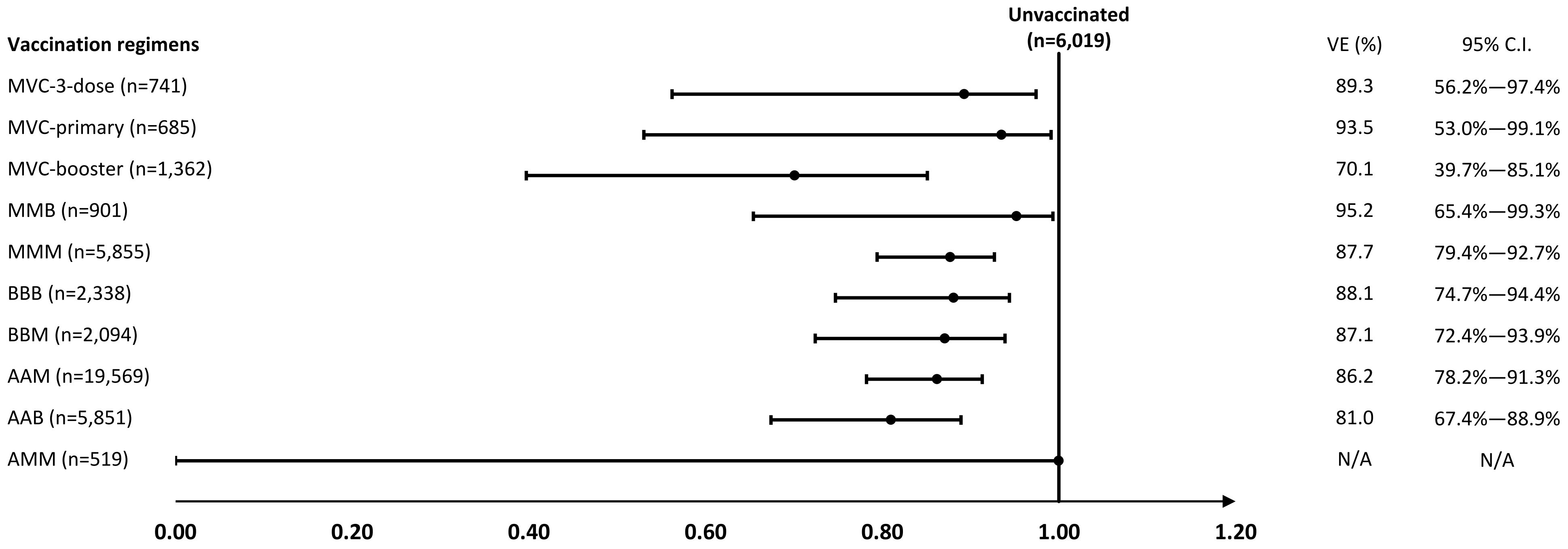

Supplementary Figure S3B. Forest plot of vaccine effectiveness against COVID-19 associated moderate to severe disease in persons 50 to 64 years old.

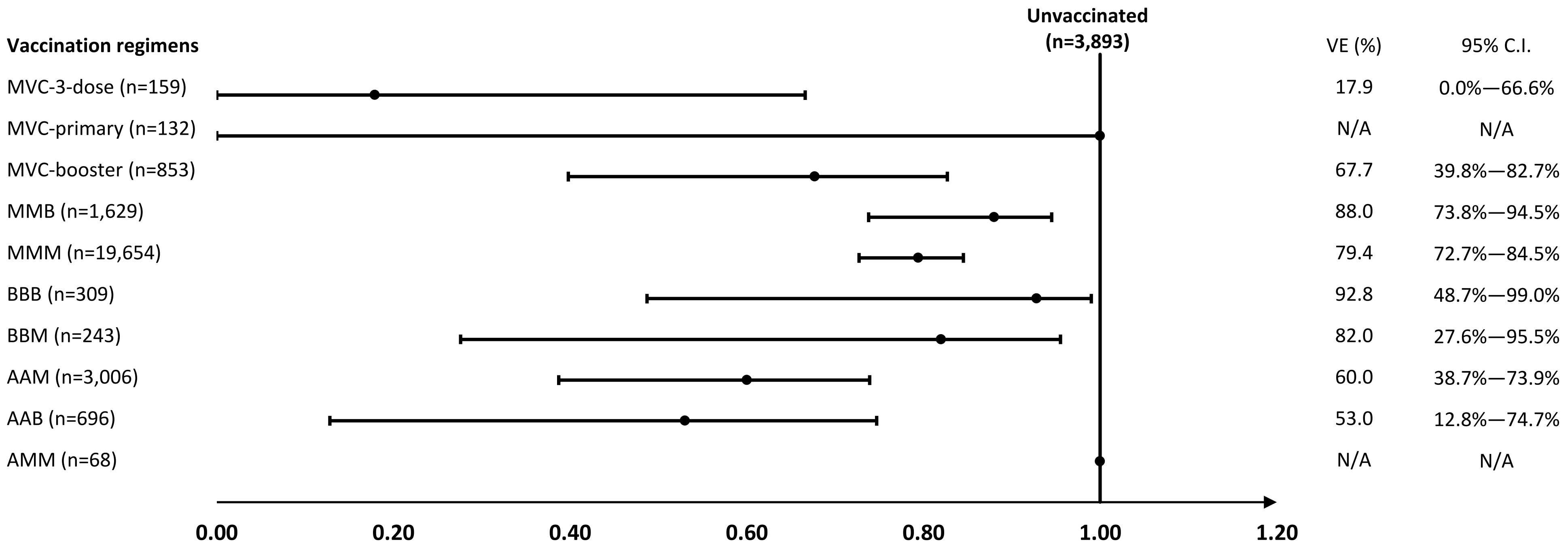

Supplementary Figure S3C. Forest plot of vaccine effectiveness against COVID-19 associated moderate to severe disease in persons 65 to 74 years old.

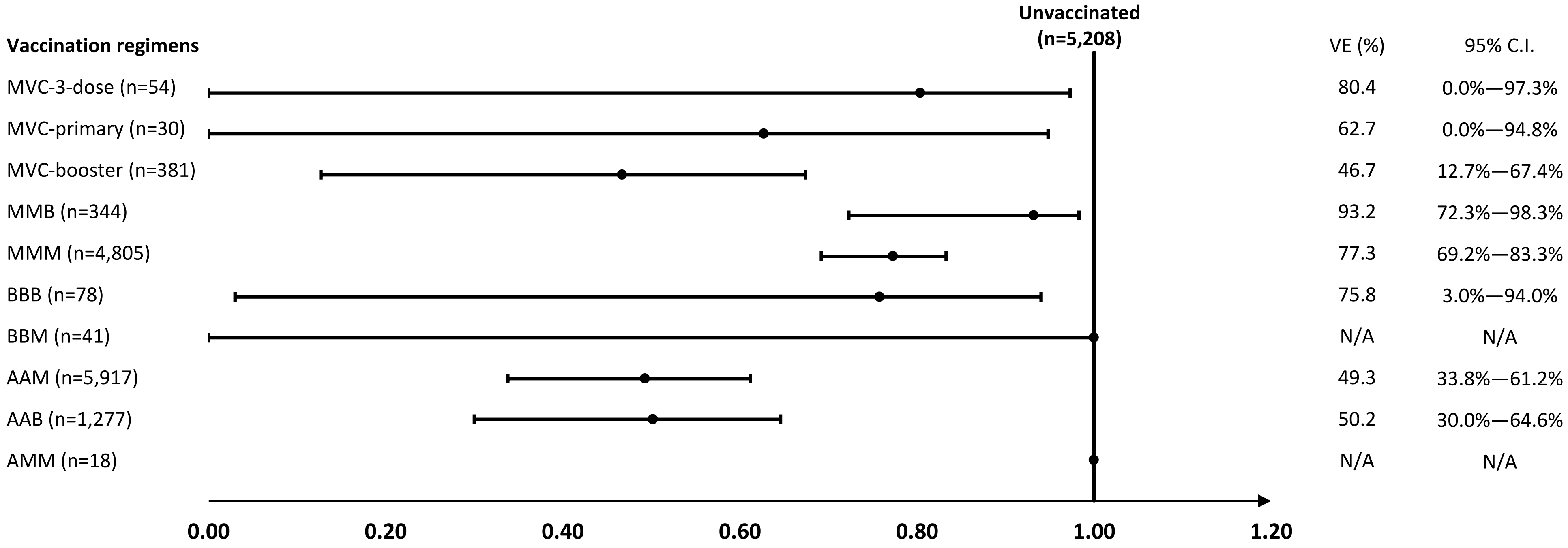

Supplementary Figure S3D. Forest plot of vaccine effectiveness against COVID-19 associated moderate to severe disease in persons > 75 years old.

Supplementary Tables

Supplementary Table S1. Age distribution of each group.

Vaccine group	Total vaccinees	Mean	SD	20—49	%	50—64	%	65—74	%	Above 75	%
Unvaccinated	25,303	56.12	19.40	10,183	40.24	6,019	23.79	3,893	15.39	5,208	20.58
MVC-3-dose[a]	1,855	49.93	13.35	901	48.57	741	39.95	159	8.57	54	2.91
MVC-primary[b]	2,892	42.27	13.22	2,045	70.71	685	23.69	132	4.56	30	1.04
MVC-booster[c]	4,233	55.16	14.83	1,637	38.67	1,362	32.18	853	20.15	381	9.00
MMB	4,035	58.00	15.78	1,161	28.77	901	22.33	1,629	40.37	344	8.53
MMM	36,239	63.44	13.83	5,925	16.35	5,855	16.16	19,654	54.23	4,805	13.26
BBB	6,486	45.91	13.46	3,761	57.99	2,338	36.05	309	4.76	78	1.20
BBM	6,166	44.97	12.78	3,788	61.43	2,094	33.96	243	3.94	41	0.66
AAM	55,299	50.80	16.30	26,807	48.48	19,569	35.39	3,006	5.44	5,917	10.70
AAB	17,141	49.09	15.01	9,317	54.36	5,851	34.13	696	4.06	1,277	7.45
AMM	2,570	40.74	12.21	1,965	76.46	519	20.19	68	2.65	18	0.70
Total	162,219	53.83	16.94	67,490	41.60	45,934	28.32	30,642	18.89	18,153	11.19

Abbreviation: SD, standard deviation; A, ChAdOx1 nCOV-19; M, mRNA-1273; B, BNT162b2; MVC, MCV-COV1901

[a] MVC-3-dose, vaccinees received 3 doses of MCV-COV1901;

[b] MVC-primary, vaccinees received 2 doses of MCV-COV1901 as primary series;

[c] MVC-booster, vaccinees received MCV-COV1901 as the booster.

Supplementary Table S2. Interval between the days when vaccinees received the 3rd dose of COVID-19 to 1st March, 2022.

Vaccine group	Total	Mean	SD	<4 weeks	%	4–8 weeks	%	>8 weeks	%
MVC-3-dose[a]	1,855	4.07	2.08	916	49.38	936	50.46	3	0.16
MVC-primary[b]	2,892	4.95	2.48	875	30.26	1,984	68.60	33	1.14
MVC-booster[c]	4,233	4.40	2.29	1,888	44.60	2,233	52.75	112	2.65
MMB	4,035	4.61	1.97	1,462	36.23	2,522	62.50	51	1.26
MMM	36,239	5.10	2.29	10,887	30.04	23,676	65.33	1,676	4.62
BBB	6,486	1.95	1.88	6,162	95.00	245	3.78	79	1.22
BBM	6,166	1.73	1.14	6,004	97.37	155	2.51	7	0.11
AAM	55,299	5.61	2.37	13,830	25.01	38,188	69.06	3,281	5.93
AAB	17,141	4.70	2.08	6,103	35.60	10,761	62.78	277	1.62
AMM	2,570	4.85	2.61	950	36.96	1,607	62.53	13	0.51
Total	162,219	4.90	2.47	74,380	45.85	82,307	50.74	5,532	3.41

Abbreviation: SD, standard deviation; A, ChAdOx1 nCOV-19; M, mRNA-1273; B, BNT162b2; MVC, MCV-COV1901

[a] MVC-3-dose, vaccinees received 3 doses of MCV-COV1901;

[b] MVC-primary, vaccinees received 2 doses of MCV-COV1901 as primary series;

[c] MVC-booster, vaccinees received MCV-COV1901 as the booster.

Supplementary Table S3. Demographics between the vaccinated and unvaccinated groups.

Characteristic	Total (N=162,219)		Unvaccinated (n=25,303)		Vaccinated (n=136,916)		ASMD
	N	%	N	%	N	%	
Age group							0.193
20–49	67,490	41.60	10,183	40.24	57,307	41.86	
50–64	45,934	28.32	6,019	23.79	39,915	29.15	
65–74	30,642	18.89	3,893	15.39	26,749	19.54	
75+	18,153	11.19	5208	20.58	12,945	9.45	
Sex							0.044
Female	92,341	56.92	13,938	55.08	78,403	57.26	
Male	69,878	43.08	11,365	44.92	58,513	42.74	
Region							0.019
South[a]	66,861	41.22	10,226	40.41	56,635	41.36	
North[b]	95,358	58.78	15,077	59.59	80,281	58.64	
Major illness[c]							0.588
without	141,067	86.96	17,870	70.62	123,197	89.98	
with	21,152	13.04	7,433	29.38	13,719	10.02	

Abbreviation: ASMD, absolute standardized mean difference

[a] Persons enrolled from southern Taiwan (Kaohsiung, Yunlin, and Chiayi)

[b] Persons enrolled from northern Taiwan (Kee-lung, Taipei, New Taipei City, Taoyuan)

[c] Subjects who had major illness according to the National Health Insurance Administration

[d] The duration between the days when vaccinees received the 3rd dose of COVID-19 vaccine and 1st March, 2022

Supplementary Table S4A. Estimated hazard ratio of infection and vaccine effectiveness against SARS-CoV-2 infection

Vaccine Group	HR	95% CI	VE (%)	95% CI	*p*-value
**unvaccinated**	1.00		0.0		
**MVC-3-dose**[a]	0.55	0.49–0.62	44.8	37.9%–51.0%	<.0001
**MVC-primary**[b]	0.62	0.57–0.67	38.3	32.6%–43.5%	<.0001
**MVC-booster**[c]	0.60	0.55–0.65	39.9	34.7%–44.6%	<.0001
**MMB**	0.52	0.48–0.57	47.7	42.9%–52.2%	<.0001
**MMM**	0.56	0.53–0.58	44.5	42.1%–46.8%	<.0001
**BBB**	0.54	0.50–0.57	46.3	42.9%–49.6%	<.0001
**BBM**	0.51	0.48–0.54	49.0	45.7%–52.2%	<.0001
**AAM**	0.58	0.55–0.60	42.4	40.2%–44.6%	<.0001
**AAB**	0.57	0.55–0.60	42.7	40.0%–45.4%	<.0001
**AMM**	0.60	0.55–0.66	39.9	33.9%–45.4%	<.0001

Abbreviation: HR, hazard ratios; CI, confidence interval; VE, vaccine effectiveness; A, ChAdOx1 nCOV-19; M, mRNA-1273; B, BNT162b2; MVC, MVC-COV1901

[a] MVC-3-dose, vaccinees received 3 doses of MVC-COV1901;

[b] MVC-primary, vaccinees received 2 doses of MVC-COV1901 as primary series;

[c] MVC-booster, vaccinees received MVC-COV1901 as the booster.

Supplementary Table S4B. Estimated hazard ratio of infection and vaccine effectiveness against COVID-19 associated moderate to severe disease.

Vaccine Group	HR	95% CI	VE (%)	95% CI	*p*-value
**unvaccinated**	1.00		0.0		
**MVC-3-dose**[a]	0.30	0.16–0.54	70.1	45.6%–83.6%	<.0001
**MVC-primary**[b]	0.20	0.10–0.43	79.6	56.7%–90.4%	<.0001
**MVC-booster**[c]	0.34	0.25–0.47	66.1	52.9%–75.5%	<.0001
**MMB**	0.10	0.06–0.17	90.3	82.6%–94.5%	<.0001
**MMM**	0.19	0.16–0.23	80.8	77.1%–83.8%	<.0001
**BBB**	0.18	0.11–0.28	82.5	72.4%–88.9%	<.0001
**BBM**	0.17	0.10–0.27	83.5	72.9%–90.0%	<.0001
**AAM**	0.30	0.25–0.36	69.7	63.9%–74.6%	<.0001
**AAB**	0.30	0.24–0.38	69.9	61.9%–76.2%	<.0001
**AMM**	0.09	0.02–0.35	91.4	65.5%–97.9%	<.0001

Abbreviation: HR, hazard ratios; CI, confidence interval; VE, vaccine effectiveness; A, ChAdOx1 nCOV-19; M, mRNA-1273; B, BNT162b2; MVC, MVC-COV1901

[a] MVC-3-dose, vaccinees received 3 doses of MVC-COV1901;

[b] MVC-primary, vaccinees received 2 doses of MVC-COV1901as primary series;

[c] MVC-booster, vaccinees received MVC-COV1901 as the booster.

Supplementary Table S5A. Estimated hazard ratio of infection and vaccine effectiveness against SARS-CoV-2 infection in persons without major illness.

Vaccine Group	HR	95% CI	VE (%)	95% CI	*p*-value
**unvaccinated**	1.00		0.0		
**MVC-3-dose**[a]	0.56	0.50–0.63	44.0	36.7%–50.5%	<.0001
**MVC-primary**[b]	0.62	0.57–0.68	37.8	31.9%–43.1%	<.0001
**MVC-booster**[c]	0.61	0.56–0.66	39.2	33.7%–44.3%	<.0001
**MMB**	0.52	0.48–0.57	47.7	42.6%–52.4%	<.0001
**MMM**	0.57	0.55–0.60	43.0	40.3%–45.5%	<.0001
**BBB**	0.54	0.51–0.58	46.0	42.3%–49.4%	<.0001
**BBM**	0.52	0.49–0.56	48.0	44.5%–51.3%	<.0001
**AAM**	0.58	0.56–0.61	41.7	39.3%–44.0%	<.0001
**AAB**	0.58	0.55–0.61	42.2	39.3%–45.0%	<.0001
**AMM**	0.60	0.54–0.66	40.3	34.1%–45.8%	<.0001

Abbreviation: HR, hazard ratios; CI, confidence interval; VE, vaccine effectiveness; A, ChAdOx1 nCOV-19; M, mRNA-1273; B, BNT162b2; MVC, MVC-COV1901

[a] MVC-3-dose, vaccinees received 3 doses of MVC-COV1901;

[b] MVC-primary, vaccinees received 2 doses of MVC-COV1901as primary series;

[c] MVC-booster, vaccinees received MVC-COV1901 as the booster.

Supplementary Table S5B. Estimated hazard ratio of infection and vaccine effectiveness against SARS-CoV-2 infection in persons with major illness.

Vaccine Group	HR	95% CI	VE (%)	95% CI	*p*-value
**unvaccinated**	1.00		0.0		
**MVC-3-dose[a]**	0.50	0.32–0.78	50.2	22.2%–68.2%	0.00
**MVC-primary[b]**	0.53	0.32–0.88	46.9	12.0%–67.9%	0.01
**MVC-booster[c]**	0.60	0.45–0.78	40.4	21.7%–54.6%	0.00
**MMB**	0.54	0.41–0.72	46.1	28.5%–59.4%	<.0001
**MMM**	0.45	0.39–0.51	55.5	48.8%–61.3	<.0001
**BBB**	0.61	0.48–0.78	38.9	21.9%–52.1%	<.0001
**BBM**	0.39	0.28–0.54	61.4	46.1%–72.3%	<.0001
**AAM**	0.49	0.42–0.57	51.4	43.4%–58.4%	<.0001
**AAB**	0.51	0.42–0.63	48.7	37.1%–58.1%	<.0001
**AMM**	0.77	0.42–1.40	23.4	0.0%–58.0%	0.38

Abbreviation: HR, hazard ratios; CI, confidence interval; VE, vaccine effectiveness; A, ChAdOx1 nCOV-19; M, mRNA-1273; B, BNT162b2; MVC, MVC-COV1901

[a] MVC-3-dose, vaccinees received 3 doses of MVC-COV1901;

[b] MVC-primary, vaccinees received 2 doses of MVC-COV1901as primary series;

[c] MVC-booster, vaccinees received MVC-COV1901 as the booster.

Supplementary Table S5C. Estimated hazard ratio of infection and vaccine effectiveness against COVID-19 associated moderate to severe disease in persons without major illness.

Vaccine Group	HR	95% CI	VE (%)	95% CI	*p*-value
**Unvaccinated**	1.00		0.0		
**MVC-3-dose**[a]	0.28	0.13–0.59	72.0	40.6%–86.8%	0.00
**MVC-primary**[b]	0.19	0.08–0.46	81.4	54.5%–92.4%	0.00
**MVC-booster**[c]	0.37	0.26–0.53	63.1	46.9%–74.4%	<.0001
**MMB**	0.08	0.04–0.16	92.5	84.1%–96.5%	<.0001
**MMM**	0.18	0.15–0.22	82.2	78.1%–85.5%	<.0001
**BBB**	0.10	0.05–0.20	90.2	80.3%–95.1%	<.0001
**BBM**	0.14	0.07–0.26	86.3	74.2%–92.7%	<.0001
**AAM**	0.30	0.24–0.37	70.2	63.4%–75.7%	<.0001
**AAB**	0.31	0.24–0.41	68.9	59.2%–76.3%	<.0001
**AMM**	0.11	0.03–0.44	89.2	56.5%–97.3%	0.00

Abbreviation: HR, hazard ratios; CI, confidence interval; VE, vaccine effectiveness; A, ChAdOx1 nCOV-19; M, mRNA-1273; B, BNT162b2; MVC, MVC-COV1901

[a] MVC-3-dose, vaccinees received 3 doses of MVC-COV1901;

[b] MVC-primary, vaccinees received 2 doses of MVC-COV1901as primary series;

[c] MVC-booster, vaccinees received MVC-COV1901 as the booster.

Supplementary Table S5D. Estimated hazard ratio of infection and vaccine effectiveness against COVID-19 associated moderate to severe disease in persons with major illness.

Vaccine Group	HR	95% CI	VE (%)	95% CI	*p*-value
**Unvaccinated**	1.00		0.0		
**MVC-3-dose[a]**	0.39	0.14–1.04	61.5	0.0%–85.8%	0.06
**MVC-primary[b]**	0.37	0.09–1.51	63.3	0.0%–91.0%	0.16
**MVC-booster[c]**	0.27	0.12–0.57	73.4	43.0%–87.6%	0.00
**MMB**	0.19	0.08–0.46	81.4	54.0%–92.5%	0.00
**MMM**	0.26	0.19–0.36	74.1	64.5%–81.2%	<.0001
**BBB**	0.44	0.24–0.81	55.7	18.8%–75.8%	0.01
**BBM**	0.29	0.13–0.66	70.8	34.4%–87.0%	0.00
**AAM**	0.38	0.27–0.53	62.3	46.7%–73.3%	<.0001
**AAB**	0.33	0.20–0.54	67.2	46.2%–80.0%	<.0001
**AMM**	0.00	$0.00–8\times10^{255}$	100.0	0.0%–100.0%	0.97

Abbreviation: HR, hazard ratios; CI, confidence interval; VE, vaccine effectiveness; A, ChAdOx1 nCOV-19; M, mRNA-1273; B, BNT162b2; MVC, MVC-COV1901

[a] MVC-3-dose, vaccinees received 3 doses of MVC-COV1901;

[b] MVC-primary, vaccinees received 2 doses of MVC-COV1901as primary series;

[c] MVC-booster, vaccinees received MVC-COV1901 as the booster.

Supplementary Table S6A. Estimated hazard ratio of infection and vaccine effectiveness against SARS-CoV-2 infection in persons 20 to 49 years old.

Vaccine Group	HR	95% CI	VE (%)	95% CI	*p*-value
**Unvaccinated**	1.00		0.0		
**MVC-3-dose**[a]	0.57	0.48–0.66	43.5	34.1%–51.6%	<.0001
**MVC-primary**[b]	0.67	0.61–0.74	32.7	25.7%–39.1%	<.0001
**MVC-booster**[c]	0.57	0.51–0.64	43.0	35.8%–49.4%	<.0001
**MMB**	0.58	0.51–0.67	41.8	33.5%–49.0%	<.0001
**MMM**	0.62	0.58–0.66	38.1	33.6%–42.2%	<.0001
**BBB**	0.55	0.51–0.60	44.8	40.4%–48.9%	<.0001
**BBM**	0.52	0.48–0.56	47.9	43.8%–51.7%	<.0001
**AAM**	0.57	0.55–0.60	42.6	39.8%–45.3%	<.0001
**AAB**	0.56	0.53–0.60	43.6	40.1%–46.9%	<.0001
**AMM**	0.63	0.57–0.70	37.1	30.1%–43.4%	<.0001

Abbreviation: HR, hazard ratios; CI, confidence interval; VE, vaccine effectiveness; A, ChAdOx1 nCOV-19; M, mRNA-1273; B, BNT162b2; MVC, MVC-COV1901

[a] MVC-3-dose, vaccinees received 3 doses of MVC-COV1901;

[b] MVC-primary, vaccinees received 2 doses of MVC-COV1901 as primary series;

[c] MVC-booster, vaccinees received MVC-COV1901 as the booster

Supplementary Table S6B. Estimated hazard ratio of infection and vaccine effectiveness against SARS-CoV-2 infection in persons 50 to 64 years old.

Vaccine Group	HR	95% CI	VE (%)	95% CI	*p*-value
**Unvaccinated**	1.00		0.0		
**MVC-3-dose[a]**	0.60	0.48–0.75	40.0	25.0%–52.0%	<.0001
**MVC-primary[b]**	0.63	0.51–0.79	36.6	20.9%–49.3%	<.0001
**MVC-booster[c]**	0.71	0.60–0.83	29.5	16.9%–40.2%	<.0001
**MMB**	0.73	0.61–0.88	26.6	11.6%–39.1%	0.00
**MMM**	0.72	0.65–0.79	28.5	21.4%–35.0%	<.0001
**BBB**	0.70	0.61–0.79	30.5	21.3%–38.7%	<.0001
**BBM**	0.63	0.55–0.72	37.4	28.5%–45.2%	<.0001
**AAM**	0.66	0.60–0.72	34.1	27.9%–39.9%	<.0001
**AAB**	0.68	0.61–0.76	32.0	24.3%–38.8%	<.0001
**AMM**	0.67	0.52–0.87	32.8	12.7%–48.2%	0.00

Abbreviation: HR, hazard ratios; CI, confidence interval; VE, vaccine effectiveness; A, ChAdOx1 nCOV-19; M, mRNA-1273; B, BNT162b2; MVC, MVC-COV1901

[a] MVC-3-dose, vaccinees received 3 doses of MVC-COV1901;

[b] MVC-primary, vaccinees received 2 doses of MVC-COV1901 as primary series;

[c] MVC-booster, vaccinees received MVC-COV1901 as the booster

Supplementary Table S6C. Estimated hazard ratio of infection and vaccine effectiveness against SARS-CoV-2 infection in persons 65 to 74 years old.

Vaccine Group	HR	95% CI	VE (%)	95% CI	*p*-value
**Unvaccinated**	1.00		0.0		
**MVC-3-dose**[a]	0.73	0.48–1.12	26.7	0.0%–52.3%	0.15
**MVC-primary**[b]	0.50	0.30–0.84	50.1	16.4%–70.2%	0.01
**MVC-booster**[c]	0.60	0.49–0.74	40.0	25.9%–51.5%	<.0001
**MMB**	0.41	0.34–0.49	59.3	50.7%–66.3%	<.0001
**MMM**	0.49	0.44–0.55	50.6	44.9%–55.7%	<.0001
**BBB**	0.47	0.33–0.68	53.1	32.4%–67.5%	<.0001
**BBM**	0.51	0.35–0.76	48.8	24.3%–65.4%	0.00
**AAM**	0.65	0.56–0.75	35.2	24.9%–44.1%	<.0001
**AAB**	0.61	0.48–0.76	39.5	24.5%–51.6%	<.0001
**AMM**	0.55	0.28–1.07	45.0	0.0%–71.7%	0.08

Abbreviation: HR, hazard ratios; CI, confidence interval; VE, vaccine effectiveness; A, ChAdOx1 nCOV-19; M, mRNA-1273; B, BNT162b2; MVC, MVC-COV1901

[a] MVC-3-dose, vaccinees received 3 doses of MVC-COV1901;

[b] MVC-primary, vaccinees received 2 doses of MVC-COV1901 as primary series;

[c] MVC-booster, vaccinees received MVC-COV1901 as the booster

Supplementary Table S6D. Estimated hazard ratio of infection and vaccine effectiveness against SARS-CoV-2 infection in persons > 75 years old.

Vaccine Group	HR	95% CI	VE (%)	95% CI	p-value
**Unvaccinated**	1.00		0.0		
**MVC-3-dose[a]**	0.66	0.33–1.32	34.3	0.0%–67.3%	0.24
**MVC-primary[b]**	0.56	0.21–1.50	44.1	0.0%–79.2%	0.25
**MVC-booster[c]**	0.70	0.53–0.93	29.6	7.0%–46.7%	0.01
**MMB**	0.46	0.32–0.65	54.0	34.7%–67.6%	<.0001
**MMM**	0.52	0.45–0.61	48.0	39.2%–55.5%	<.0001
**BBB**	0.38	0.18–0.80	62.1	20.1%–82.0%	0.01
**BBM**	0.12	0.02–0.84	88.2	16.4%–98.3%	0.03
**AAM**	0.68	0.58–0.79	32.2	21.0%–41.7%	<.0001
**AAB**	0.64	0.53–0.77	36.2	22.6%–47.4%	<.0001
**AMM**	0.52	0.13–2.07	48.5	0.0%–87.2%	0.35

Abbreviation: HR, hazard ratios; CI, confidence interval; VE, vaccine effectiveness; A, ChAdOx1 nCOV-19; M, mRNA-1273; B, BNT162b2; MVC, MVC-COV1901

[a] MVC-3-dose, vaccinees received 3 doses of MVC-COV1901;

[b] MVC-primary, vaccinees received 2 doses of MVC-COV1901 as primary series;

[c] MVC-booster, vaccinees received MVC-COV1901 as the booster

Supplementary Table S7A. Estimated hazard ratio of infection and vaccine effectiveness against COVID-19 associated moderate to severe disease in persons 20 to 49 years old.

Vaccine Group	HR	95% CI	VE (%)	95% CI	*p*-value
**Unvaccinated**	1.00		0.0		
**MVC-3-dose**[a]	0.41	0.13–1.33	58.7	0.0%–87.2%	0.14
**MVC-primary**[b]	0.33	0.13–0.85	67.1	15.0%–87.2%	0.02
**MVC-booster**[c]	0.15	0.04–0.60	85.3	39.6%–96.4%	0.01
**MMB**	0.24	0.06–1.00	76.1	0.4%–94.3%	0.05
**MMM**	0.39	0.21–0.72	61.2	28.2%–79.0%	0.00
**BBB**	0.26	0.13–0.52	73.7	47.9%–86.7%	0.00
**BBM**	0.22	0.10–0.46	78.5	53.7%–90.0%	<.0001
**AAM**	0.23	0.14–0.36	77.3	64.4%–85.6%	<.0001
**AAB**	0.20	0.11–0.36	80.2	63.8%–89.2%	<.0001
**AMM**	0.17	0.04–0.70	83.2	30.3%–96.0%	0.01

Abbreviation: HR, hazard ratios; CI, confidence interval; VE, vaccine effectiveness; A, ChAdOx1 nCOV-19; M, mRNA-1273; B, BNT162b2; MVC, MVC-COV1901

[a] MVC-3-dose, vaccinees received 3 doses of MVC-COV1901;

[b] MVC-primary, vaccinees received 2 doses of MVC-COV1901 as primary series;

[c] MVC-booster, vaccinees received MVC

Supplementary Table S7B. Estimated hazard ratio of infection and vaccine effectiveness against COVID-19 associated moderate to severe disease in persons 50 to 64 years old.

Vaccine Group	HR	95% CI	VE (%)	95% CI	*p*-value
**Unvaccinated**	1.00		0.0		
**MVC-3-dose**[a]	0.11	0.03–0.44	89.3	56.2%–97.4%	0.00
**MVC-primary**[b]	0.07	0.01–0.47	93.5	53.0%–99.1%	0.01
**MVC-booster**[c]	0.30	0.15–0.60	70.1	39.7%–85.1%	0.00
**MMB**	0.05	0.01–0.35	95.2	65.4%–99.3%	0.00
**MMM**	0.12	0.07–0.21	87.7	79.4%–92.7%	<.0001
**BBB**	0.12	0.06–0.25	88.1	74.7%–94.4%	<.0001
**BBM**	0.13	0.06–0.28	87.1	72.4%–93.9%	<.0001
**AAM**	0.14	0.09–0.22	86.2	78.2%–91.3%	<.0001
**AAB**	0.19	0.11–0.33	81.0	67.4%–88.9%	<.0001
**AMM**	0.00	0.00–6.93×10^{185}	100.0	0.0%–100%	0.95

Abbreviation: HR, hazard ratios; CI, confidence interval; VE, vaccine effectiveness; A, ChAdOx1 nCOV-19; M, mRNA-1273; B, BNT162b2; MVC, MVC-COV1901

[a] MVC-3-dose, vaccinees received 3 doses of MVC-COV1901;

[b] MVC-primary, vaccinees received 2 doses of MVC-COV1901 as primary series;

[c] MVC-booster, vaccinees received MVC-COV1901 as the booster

Supplementary Table S7C. Estimated hazard ratio of infection and vaccine effectiveness against COVID-19 associated moderate to severe disease in persons 65 to 74 years old.

Vaccine Group	HR	95% CI	VE (%)	95% CI	*p*-value
**Unvaccinated**	1.00		0.0		
**MVC-3-dose**[a]	0.82	0.33–2.02	17.9	0.0%–66.6%	0.67
**MVC-primary**[b]	0.00	0.00–5.21×10^{279}	100	0.0%–100.0%	0.97
**MVC-booster**[c]	0.32	0.17–0.60	67.7	39.8%–82.7%	0.00
**MMB**	0.12	0.06–0.26	88.0	73.8%–94.5%	<.0001
**MMM**	0.21	0.16–0.27	79.4	72.7%–84.5%	<.0001
**BBB**	0.07	0.01–0.51	92.8	48.7%–99.0%	0.01
**BBM**	0.18	0.05–0.72	82.0	27.6%–95.5%	0.02
**AAM**	0.40	0.26–0.61	60.0	38.7%–73.9%	<.0001
**AAB**	0.47	0.25–0.87	53.0	12.8%–74.7%	0.02
**AMM**	N/A	N/A	N/A	N/A	N/A

Abbreviation: HR, hazard ratios; CI, confidence interval; VE, vaccine effectiveness; A, ChAdOx1 nCOV-19; M, mRNA-1273; B, BNT162b2; MVC, MVC-COV1901

[a] MVC-3-dose, vaccinees received 3 doses of MVC-COV1901;

[b] MVC-primary, vaccinees received 2 doses of MVC-COV1901 as primary series;

[c] MVC-booster, vaccinees received MVC-COV1901 as the booster

Supplementary Table S7D. Estimated hazard ratio of infection and vaccine effectiveness against COVID-19 associated moderate to severe disease in persons > 75 years old.

Vaccine Group	HR	95% CI	VE (%)	95% CI	*p*-value
**Unvaccinated**	1.00		0.0		
**MVC-3-dose**[a]	0.20	0.03–1.40	80.4	0.0%–97.3%	0.10
**MVC-primary**[b]	0.37	0.05–2.66	62.7	0.0%–94.8%	0.33
**MVC-booster**[c]	0.53	0.33–0.87	46.7	12.7%–67.4%	0.01
**MMB**	0.07	0.02–0.28	93.2	72.3%–98.3%	0.00
**MMM**	0.23	0.17–0.31	77.3	69.2%–83.3%	<.0001
**BBB**	0.24	0.06–0.97	75.8	3.0%–94.0%	0.05
**BBM**	0.00	$0.0–1.43\times10^{270}$	100.0	0.0%–100.0%	0.97
**AAM**	0.51	0.39–0.66	49.3	33.8%–61.2%	<.0001
**AAB**	0.50	0.35–0.70	50.2	30.0%–64.6%	<.0001
**AMM**	0.00	0.00–0.00	100.0	100.0%–100.0%	0.98

Abbreviation: HR, hazard ratios; CI, confidence interval; VE, vaccine effectiveness; A, ChAdOx1 nCOV-19; M, mRNA-1273; B, BNT162b2; MVC, MVC-COV1901

[a] MVC-3-dose, vaccinees received 3 doses of MVC-COV1901;

[b] MVC-primary, vaccinees received 2 doses of MVC-COV1901 as primary series;

[c] MVC-booster, vaccinees received MVC-COV1901 as the booster
